# The Nutritive Value of Rectal Feeding

**Published:** 1901-04-13

**Authors:** 


### THE NUTRITIVE VALUE OF RECTAL FEEDING.

What exactly is the nature of the benefit which is derived in many cases from rectal feeding is open to question. In some cases probably it is the watery element that is most important; in fact, considering the benefit which is derived by many patients from enemata of beef tea, the nutrient value of which, measured in calories, is practically *nil*, one may feel quite certain that rectal feeding is often merely a means of giving sufficient water to enable the patient to go on living upon his own tissues. In other cases in which care is taken to select the right material a fair amount of nutriment may be absorbed by the rectum. But at the best, as ordinarily given, rectal alimentation is a poor substitute for feeding by the mouth. Dr. Grunbaum,[1] writing on this subject, says that if nutrient enemata consisting of 60 c.cm. of milk and 60 c.cm. of beef tea be administered every four hours, along with a few minims of liquor pancreaticus, practically all the proteid and lactose is absorbed along with the greater part of the fat. Nevertheless, the diet so taken is most insufficient, the total food value, as represented in calories, not amounting to a seventh of what would be required by an average woman at rest. With the object of obtaining better results from rectal alimentation Dr. Grunbaum has been experimenting with a mixture of ox-serum, glucose, and milk, with liquor pancreaticus. This, he says, contains a constant quantity of proteid, which is easily absorbed by the mucous membrane of the large intestine, is inexpensive, and does not require any tedious preparation—simply the addition of a preservative. He has been in the habit of adding two grains of chloretone to each ounce of serum, since that drug not only acts as an antiseptic, but also as a sedative. From analyses of the material removed by the water enema, which is given every day to wash out the rectum, it was found that less than a gramme of proteid was excreted unaltered at a time when 51 grammes per day were being administered by enemata repeated every four hours. Carbohydrates in the form of starch or glucose are readily absorbed when given in solution by the rectum, and if the strength of the solution is less than 15 per cent. it is well retained and not irritating; so that 90 grammes may be introduced during the twenty-four hours. Fat is not easily absorbed, and of the 18 grammes contained in the milk some has invariably been returned in the wash-out enemata. It would appear that where it is desired to introduce nutriment by the rectum, ox-serum is a useful substitute for other forms of food, and enables us to administer a considerably larger quantity of proteid than could otherwise be got in. There is another great advantage in ox-serum over egg albumin—namely, that it does not give rise to offensive stools.

[1] Brit. Med. Jour., April 6.

